# Nesfatin-1 suppresses autophagy of chondrocytes in osteoarthritis via remodeling of cytoskeleton and inhibiting RhoA/ROCK signal pathway

**DOI:** 10.1186/s13018-023-03539-5

**Published:** 2023-03-02

**Authors:** Lifeng Jiang, Safwat Adel Abdo Moqbel, Junxiong Zhu, Qiangchang Fu, Jiabin Lai, Changjian Lin, Lidong Wu

**Affiliations:** 1 Department of Orthopedic Surgery, The Second Affiliated Hospital, Zhejiang University School of Medicine, Hangzhou, China; 2 Orthopedics Research Institute of Zhejiang University, Hangzhou, China; 3 Key Laboratory of Motor System Disease Research and Precision Therapy of Zhejiang Province, Hangzhou, China; 4 Clinical Research Center of Motor System Disease of Zhejiang Province, Hangzhou, China

**Keywords:** Nesfatin-1, Osteoarthritis, Autophagy, Cytoskeleton, RhoA/ROCK

## Abstract

Autophagy and cytoskeleton integrity of chondrocytes are a considered as major factors in the progression of osteoarthritis (OA) involving excessive chondrocyte apoptosis and senescence. Nesfatin-1, an adipokine, has been reported to be closely related to cell autophagy and cytoskeleton malfunction. Our previous study found that nesfatin-1 was highly correlated with OA progress in OA patient, and the expression of nesfatin-1 rises in knee articular tissue, serum and chondrocytes. In current study, we aimed to explore the therapeutic effect of nesfatin-1 on OA and its molecular mechanism related to chondrocyte autophagy and cytoskeleton malfunction. We firstly demonstrated that nesfatin-1 effectively suppressed excessive autophagy of OA chondrocytes at both gene and protein levels. Meanwhile, we also found that nesfatin-1 significantly improved cytoskeleton integrity by showing higher F-actin/G-actin ratio, as well as more organized actin fiber structure. Mechanistically, utility of RhoA activator and inhibitor revealed that regulation of autophagy and cytoskeleton integrity via nesfatin-1 was realized via RhoA/ROCK pathway. We also confirmed that nesfatin-1 significantly ameliorated IL-1β induced cartilage degeneration via destabilization of the medial meniscus (DMM) model. Overall, our study indicates that nesfatin-1 might be a promising therapeutic molecule for OA intervention.

## Background

Osteoarthritis (OA) has become the most common form of arthritis; however, the primary therapy for OA remains to be symptomatic alleviation through lifestyle adoption, and in more severe cases surgery. Although several pathological pathways, such as NFκB and Nrf2 pathway, have been identified to be associated with OA, structural destruction of articular cartilage is still considered to the root cause of OA. Since the integrity of articular cartilage is maintained by extracellular matrix homeostasis via chondrocyte metabolism, their dysfunctions including morphological change, genetic mutation and other physiological abnormality are closely related to OA progression [1]. Thus, further understanding of cartilage ECM and its relationship with OA progression is requisite to finding novel effective OA therapeutic strategies.

Autophagy is a form of lysosomal degradation required for the maintenance of cell and tissue homeostasis [2, 3], and overactivation of autophagy can cause cell death with unique morphological features distinct from apoptosis or necrosis [4, 5]. Autophagy-dependent cell death is characterized by its dependence on the autophagic machinery, and cell death is enhanced when feedback mechanisms to restrain autophagy are disrupted [6]. There is accumulating evidence that demonstrates the pathological relation between abnormal autophagy levels in chondrocyte and OA [7–9]. Chang et al. showed that excessive activation of autophagy led to autophagic cell death in OA chondrocytes in vitro [10]. Take into account the importance of chondrocyte autophagy during OA development, it is therefore necessary to further explore its molecular and cellular mechanism involved in OA development.

Cytoskeleton helps cells maintain their shape and internal organization, which also provides mechanical support that enables cells to carry out essential functions. Recent studies have demonstrated that morphological destruction of chondrocyte cytoskeleton is related to osteoarthritis [11, 12]. For example, Capín-Gutiérrez et al. found that the expression of cytoskeleton proteins was reduced in OA cartilage in comparison with normal cartilage by western blot and confocal analysis [11]; Jin et al. found that regulating cytoskeleton by resveratrol protects chondrocytes from apoptosis [13]. These findings suggested that destruction of chondrocyte cytoskeleton during OA progression might be related to deterioration of OA. On the other side, actin cytoskeleton dynamics is also actively involved in multiple forms of intracellular trafficking including macroautophagy. For instance, starved cells treated with actin depolymerization agents failed to generate autophagosomes. It was also found that the actin filaments were colocalized with essential autophagy markers [14, 15], suggesting that it is along the cytoskeleton that autophagosomes are pulled into lysosomes to form mature autolysosomes.

Nesfatin-1 is an 82-amino-acid peptide that was firstly described in 2006 by Oh IS and colleagues from Maebashi, Japan [16]. Nesfatin-1 is the only biologically active peptide known to be involved in food restriction, and many researchers have focused on the neuroendocrine role of nesfatin-1. Recently, more functions of nesfatin-1 have been discovered, such as anti-oxidation [17], anti-apoptosis [18] and regulation of autophagy [19]. In our previous study, we demonstrated that nesfatin-1 ameliorated OA symptoms via suppressing IL-1β-induced inflammation, apoptosis and ECM decomposition [20]. Further, we found that nesfatin-1 was capable of inhibiting excessive autophagy, which accelerated the osteogenic differentiation of tendon-derived stem cells [19].

Due to the pivotal roles of NES-1 on multiple pathological scenarios against OA, we aimed to explore the anti-overautophagy mechanism of nesfatin-1in the current study. We hypothesized that nesfatin-1 ameliorated the progression of OA by inhibiting excessive autophagy, and nesfatin-1 regulated overactivation of autophagy via ROCK-associated cytoskeletal changes. To validate this hypothesis, RT-PCR, western blot, immunofluorescence assay and immunohistochemical staining were performed to investigate the potential regulatory mechanism of nesfatin-1 on OA both in vitro and in vivo. Our data suggest that nesfatin-1 was capable to improve the course of OA through suppressing the level of excessive autophagy and restoring cytoskeleton integrity via inhibiting RhoA/ROCK pathway. In vivo experiments also verified that nesfatin-1 inhibited RhoA/ROCK pathway and thereby slowed down the progression of OA in rat model. We demonstrate that nesfatin-1 might be a novel biomolecule to develop a promising therapeutic strategy for OA patients.

## Materials and methods

### SD rats chondrocytes extraction and culture

SD rats were anesthetized and executed to remove the knee articulation tissue immediately. Then, PBS (containing 100 U/ml penicillin and streptomycin) was used to wash the extracted tissues three times. Trypsin and collagenase type II were utilized to digest tissues, following 1000 rpm centrifugation to obtain chondrocytes in the precipitate. Subsequently, chondrocytes were cultured using DMEM with 10% FBS at 37 °C, 5%CO_2_. The primary chondrocytes were allowed to passage 3 times for further experiments. IL-1β was added into chondrocytes to induce osteoarthritis (OA) for the next assay.

### Quantitative real-time PCR (qRT-PCR)

Chondrocytes were collected in logarithmic growth period, and nesfatin-1 was added at different time periods, and then, cells were mixed with TRIzol. Chloroform was added for 15-min maintenance at room temperature, and then, the solution was added with isopropyl alcohol and centrifuged to obtain the RNA sediment. Finally, the RNA extraction was dried and hoarded under − 80 °C till experiments. QRT-PCR analyses of Atg5, Beclin1 and Atg7 were performed using M-MLV reverse transcription Kit and SYBR Prime Script RT-PCR Kits based on the protocol. The transcription level was subsequently analyzed by 2^−ΔΔCt^ method.

### Western blotting

Cells were collected and lysed in lysis buffer on ice for 30 min. Then, the protein quantity was calculated using BCA Kit. Proteins solution was then added with loading buffer and boiled for 5 min. Subsequently, proteins were transferred onto polyvinyldifluoride membranes (Bio-Rad Laboratories USA) after separated on SDS-PAGE. The membranes were immersed with the diligent of LC3B, Atg5, Atg7, Beclin1, LIMK1, p-LIMK1, Cofilin-2 and p-Cofilin-2 proteins’ antibodies, while β-actin was used as internal control. Then, observation was performed through an enhanced chemiluminescence reagent (Thermo Scientific, Waltham, MA, USA).

### Immunofluorescence examination

Immunofluorescence staining was utilized to further estimate the autophagy level and cytoskeleton in chondrocytes with different treatments. Cells were cultured to reach a confluence of roughly 80% and then were divided into groups for different treatments. PBS was used to wash cells three times, and cells were fixed by cold methanol for 20 min. 0.1% Triton X-100 was utilized to permeate the fixed cells. Subsequently, the primary antibody of LC3 (Proteintech, 14600-1-AP) was used to treat cells at 4 °C for 24 h. A secondary Alexa Fluor® 594-conjugated antibody was added and maintained at 25 °C for another 1 h. G-actin was stained using G-actin red fluorescence staining kit (Chundu Biotechnology, CD-101405GM). F-actin was stained with YF Dye Phalloidin Conjugates (USEVERBRIGHT, YP0059-50UG) directly. DAPI was utilized to stain nucleus for 5 min. Finally, the fluorescence was observed with a Leitz DM40 microscope (Leica Microsystems, Wetzlar, Germany). The relative intensity was analyzed by ImageJ.

### RhoGTP activity estimation

Chondrocytes with different treatments for experiments were collected and washed using PBS. Chondrocytes were lysed with RIPA lysis buffer (Beyotime, P0013C) for 15 min and then centrifuged using 14,000 rpm at 4 °C for 5 min. The supernatant was collected and stored at − 20 °C and was used for the RhoGTP activity determination. The activity of RhoGTP was evaluated using RhoGTP ELISA Assay Kit (Nuoyuan, YX1962251, China) according to manufacturer’s protocol.

### Animal study and immunohistochemical staining (IHC)

SD rats meniscectomy model was constructed for experiment. Rats were divided into 4 groups, which are control group, IL-1β-treated group and IL-1β plus nesfatin-1 (10 ng/ml)-treated group, and each group has 5 rats. The correlated treatments were given to SD rats through intra-articular injection for 6 weeks. Then, knee articulation tissues were resected after treatment and the tissues were used for immunohistochemical staining. All tissues were hoarded under − 20 °C for 2 days. Then, tissues samples were washed with PBS and fixed using acetone for 5 min. PBS containing 0.2% Triton X-100 was utilized to re-wash the slice. Primary antibodies and correlated secondary antibodies were co-incubated with the sample slice to complete immunohistochemical staining.

### Statistical analyses

The results data were expressed as mean plus standard deviation (SD) for triplicate measurements. A two-tailed Student’s t test was employed to analyze differences between two groups. Multiple comparisons between groups were performed using analysis of variance (ANOVA) followed by Tukey’s post hoc test through SPSS (13.0) or GraphPad Prism 7.0. *p* < 0.05 means statistically significance.

## Results

### Excessive autophagy is associated with osteoarthritis development induced chondrocytes induced by IL-1β in vitro

**Fig. 1:**
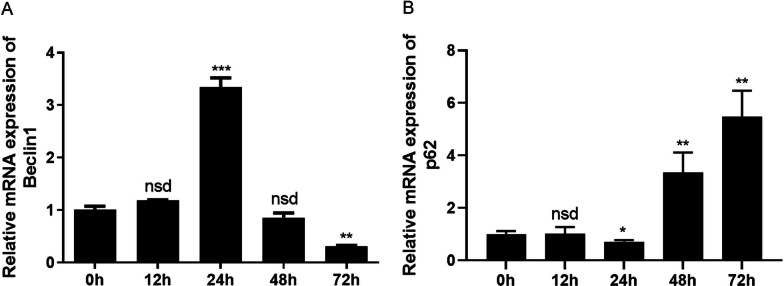
The transcription levels of autophagy related proteins in chondrocytes with IL-1β induction at different timepoints determined through qRT-PCR. The mRNA level of **A** Beclin1 and **B** p62. Data are expressed as means ± SD of 3 independent experiments. *p < 0.05, **p < 0.01, ***p < 0.001, compared to 0 h. nsd means no significant difference

**Fig. 2:**
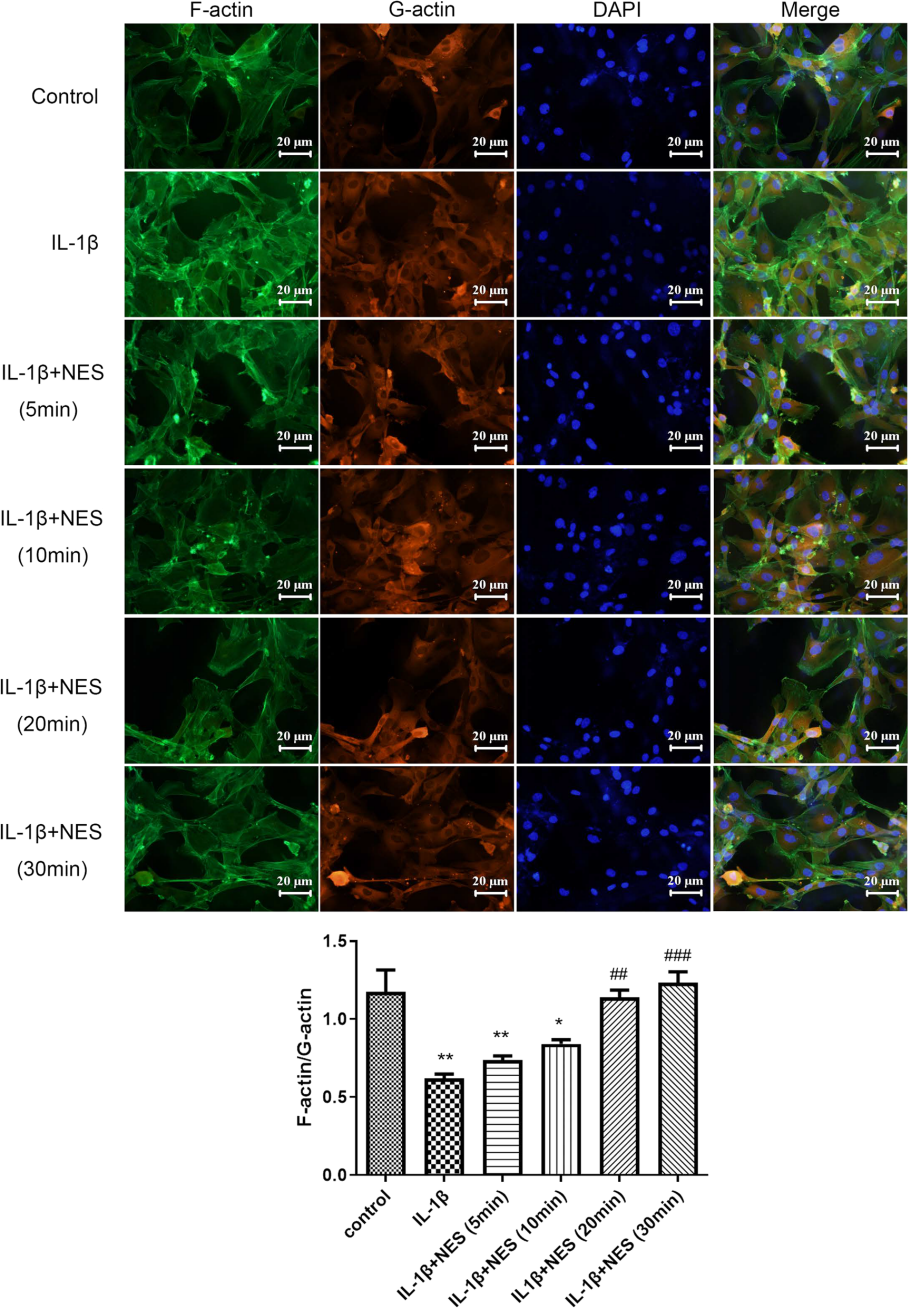
Immunofluorescence staining and quantifications of mean fluorescence intensity of F-actin and G-actin in OA chondrocytes at different time periods after 10 ng/ml of nesfatin-1 pre-treatment. The quantifications of mean fluorescence intensity were analyzed by ImageJ. Data are expressed as means ± SD of 3 independent experiments. *p < 0.05, **p < 0.01 (compared with control group). ^##^*p* < 0.01, ^###^*p* < 0.001 (compared with IL-1β group)

Inflammatory factors are known to control OA development [21]. Among the chief cytokines, interleukin-1β (IL-1β) was reported to be highly expressed in OA animals and humans, and the level of IL-1β positively correlated with the progression of disease [22]. As shown in Fig. 1A, B, after 24 h of IL-1β treatment, Beclin1 (**p < 0.01, compared to 0 h) showed the highest transcription level, while p62 showed the lowest, indicating that chondrocytes treated with IL-1β exhibit highest degrees of autophagy at 24 h. The significantly high level of autophagy at 24 h may cause irreversible damage and activate apoptosis [23], thus exacerbating OA. Autophagy was reduced at 48 h, and this was observed to be the result of compensatory behavior to counter the damage brought on by the 24 h-excessive autophagy. Subsequently, we explored whether autophagy at the 24 h timepoint could be alleviated by nesfatin-1 (Fig. 2).

### Nesfatin-1 priming suppressed excessive autophagy in OA chondrocytes

**Fig. 3:**
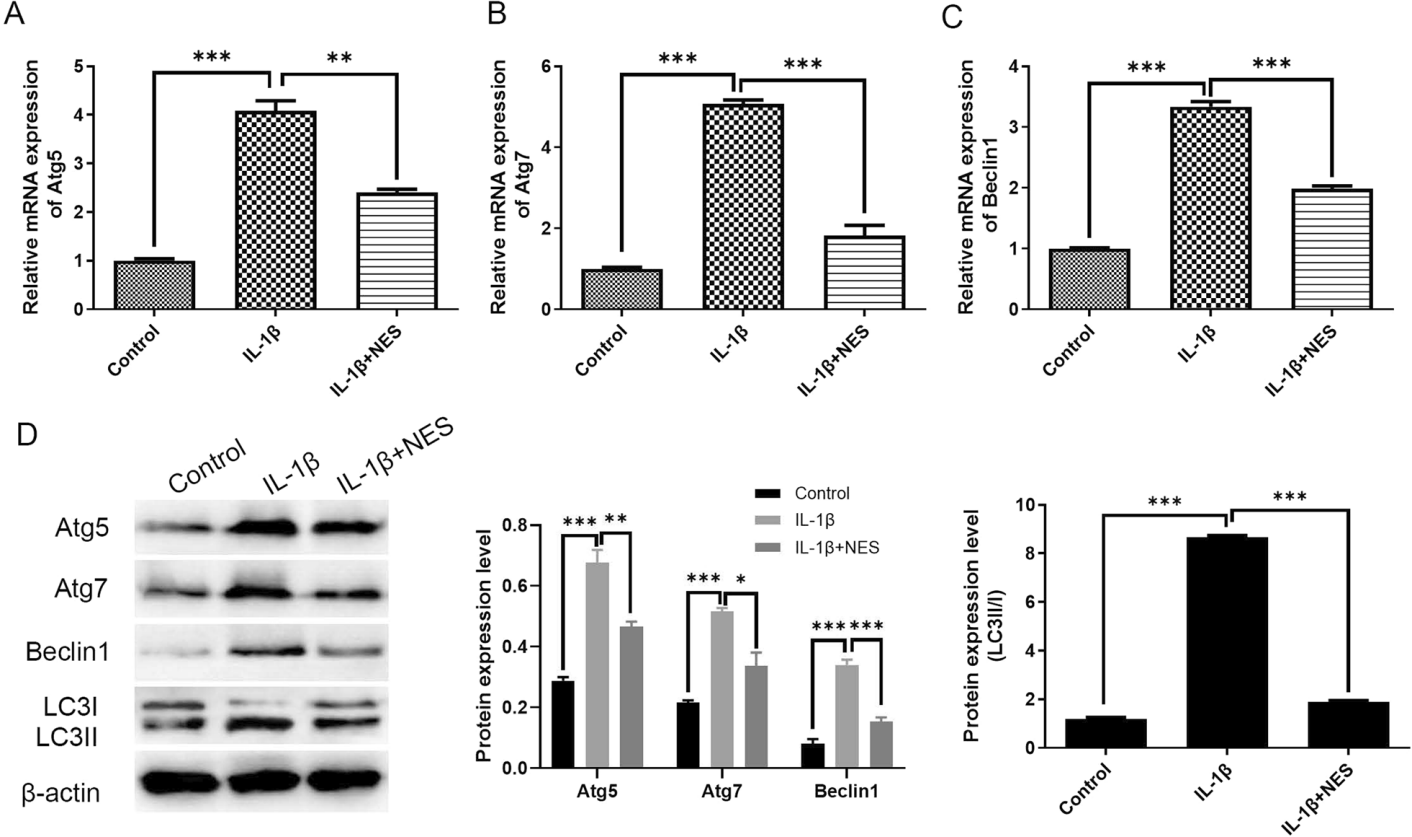
Nesfatin-1 pre-treatment conspicuously decreased the OA chondrocytes autophagy at 24 h timepoint. **A**–**C** The mRNA of Atg5, Atg7 and Beclin1 by qRT-PCR at 24 h timepoint in OA chondrocytes. **D** Western blot results demonstrated that 10 ng/ml of nesfatin-1 decreased the expression levels of autophagy correlated proteins including Atg5, Atg7, Beclin1 and LC3II/I ratio at 24 h timepoint. Data are expressed as means ± SD of 3 independent experiments. *p < 0.05, **p < 0.01, ***p < 0.001 with 0 ng/ml group; nsd means no significant difference

**Fig. 4:**
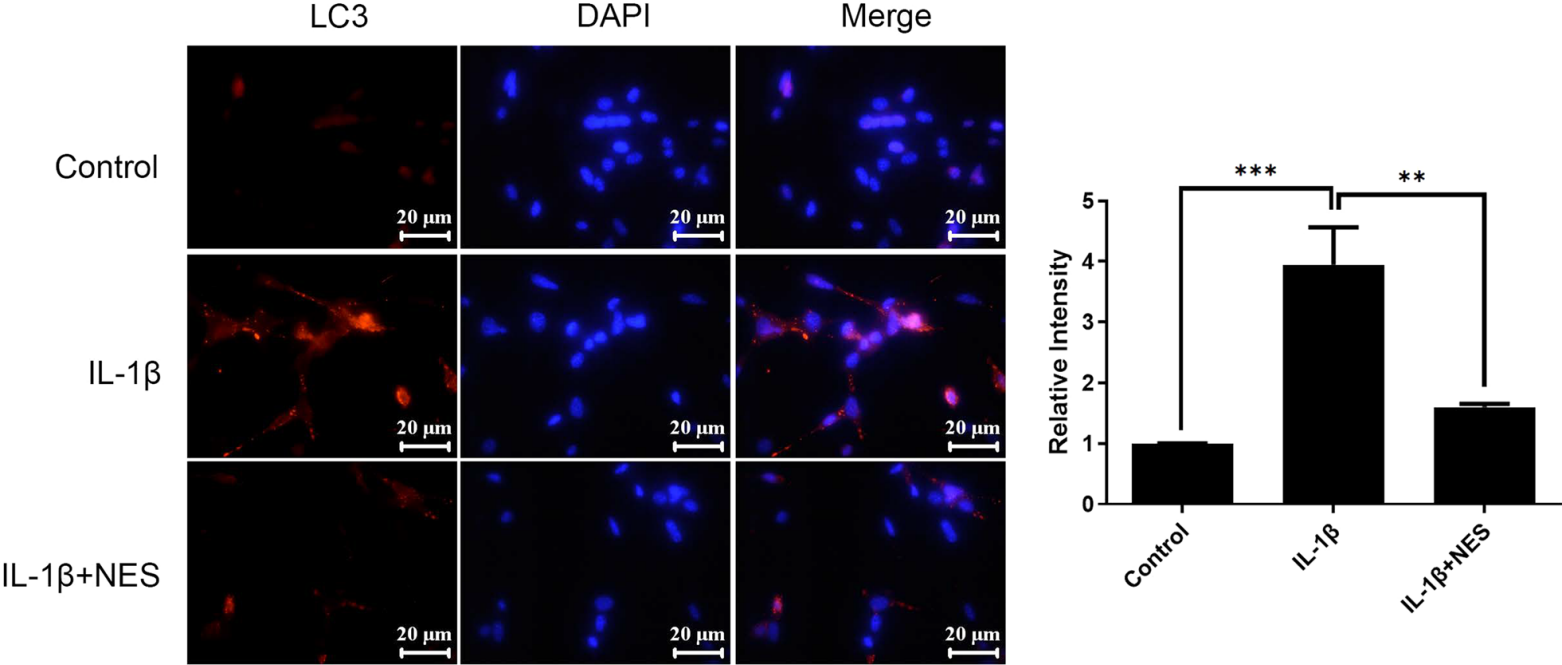
Immunofluorescence staining and quantification of mean fluorescence intensity of LC3 after 30 min pre-treatment of nesfatin-1 in OA chondrocytes at 24 h timepoint. The quantification of mean fluorescence intensity was analyzed by ImageJ. Data are expressed as means ± SD of 3 independent experiments. *p < 0.05, **p < 0.01, ***p < 0.01 compared with 0 ng/ml group

10 ng/ml of nesfatin-1 was pre-administrated to chondrocytes, and after 24 h of incubation with IL-1β, qRT-PCR and western blot were done examine the expression levels of autophagy correlated proteins in OA chondrocytes. 10 ng/ml nesfatin-1 significantly decreased the transcription level of Beclin1, Atg5 and Atg7 in OA chondrocytes (Fig. 3A–C), bringing their levels close to that of normal chondrocytes, suggesting that nesfatin-1 was indeed able to reduce autophagy in OA chondrocytes. Pre-treatment with NES significantly decreased the excessive autophagy at the 24 h timepoint in OA chondrocytes, made evident by analysis at protein level, shown in Fig. 3D. Subsequently, immunofluorescence staining on LC3 was conducted to further verify the autophagy level of OA chondrocytes with nesfatin-1 pre-treatment. As shown in Fig. 4, the fluorescence intensity of LC3 was significantly negatively regulated after pre-treatment with nesfatin-1 compared to that in OA chondrocytes without any treatment.

### Nesfatin-1 improved cytoskeleton remodeling

In accordance with our previous study [18], 10 ng/ml was selected as the optimal concentration to explore the effect of nesfatin-1 on cytoskeleton. The levels of F-actin and G-actin were stained by immunofluorescence and recorded by confocal microscopy, and the ratio of F-actin to G-actin was then analyzed quantitatively. Rat chondrocytes were pre-treated with nesfatin-1 at 5 min, 10 min, 20 min and 30 min before adding IL-1β. After 24 h-induction with IL-1β, the integrity of chondrocyte cytoskeleton was investigated, in other words the ratio of immunofluorescence intensity of F-actin and G-actin. The chondrocytes stimulated by IL-1β presented the lowest ratio with worst cytoskeleton, shown in Fig. 4. However, pre-treatment with NES for 10 min, 20 min and 30 min showed subsequent improvement in cytoskeleton integrity with increasing ratio of F-actin/G-actin, especially apparent after 30 min where integrity closely resembled normal chondrocytes (control group) (Fig. 4). From this, we deduced that 10 ng/ml of nesfatin-1 improved the integrity of OA chondrocyte cytoskeleton.

### Pre-treatment with nesfatin-1 inhibited RhoA/ROCK pathway activation in OA chondrocytes

**Fig. 5:**
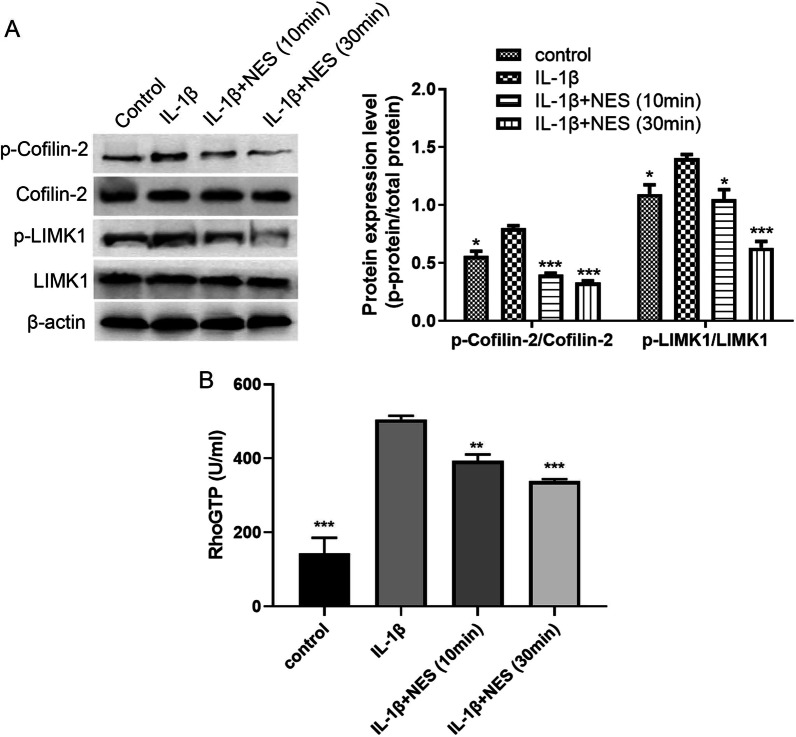
Pre-treatment with nesfatin-1 inhibited RhoA/ROCK pathway activation in OA chondrocytes. **A** The expression of RhoA/ROCK downstream proteins (Cofilin-2, p-Cofilin-2, LIMK1 and p-LIMK1) in OA chondrocytes in different groups determined by western blot and quantified by ImageJ. **B** The activity of RhoGTPase in OA chondrocytes in different groups determined by RhoGTP ELISA kit. Data are expressed as means ± SD of 3 independent experiments. *p < 0.05, **p < 0.01, ***p < 0.01 compared with IL-1β group

We hypothesized that nesfatin-1 pre-treatment could prevent the progression of OA through mediating the RhoA/ROCK pathway. We estimated the activation of the RhoA/ROCK pathway in normal and OA chondrocytes by assessing LIMK and Cofilin, downstream proteins of the RhoA/ROCK pathway. Their phosphorylation level represents the extent of RhoA/ROCK pathway activation [24]. As shown in Fig. 5A, the phosphorylation levels of Cofilin-2 and LIMK1 in OA chondrocytes were significantly higher than those in normal chondrocytes. The phosphorylation levels of Cofilin-2 and LIMK1 in OA chondrocytes when pre-treated with nesfatin-1 were significantly decreased compared to OA chondrocytes without nesfatin-1 pre-treatment (Fig. 5A). These results indicate that nesfatin-1 exerted a presumably preventative function against OA via precluding the activation of the RhoA/ROCK pathway. Furthermore, the activity of RhoGTP contents was determined based on the kit. Compared to normal chondrocytes (control), RhoGTP level in OA chondrocytes (IL-1β group) was the highest, while the OA chondrocytes with 30-min of nesfatin-1 treatment showed significantly lower RhoGTP level and weaker RhoGTP activity compared with IL-1β group (Fig. 5B). This strongly suggests that nesfatin-1 ameliorated OA through inhibiting the activity of RhoGTP. Altogether, pre-treatment with nesfatin-1 inhibited RhoA/ROCK pathway activation in OA chondrocytes.

### Blocking of RhoA/ROCK pathway improved cytoskeleton integrity and suppressed excessive autophagy level in OA chondrocytes

**Fig. 6:**
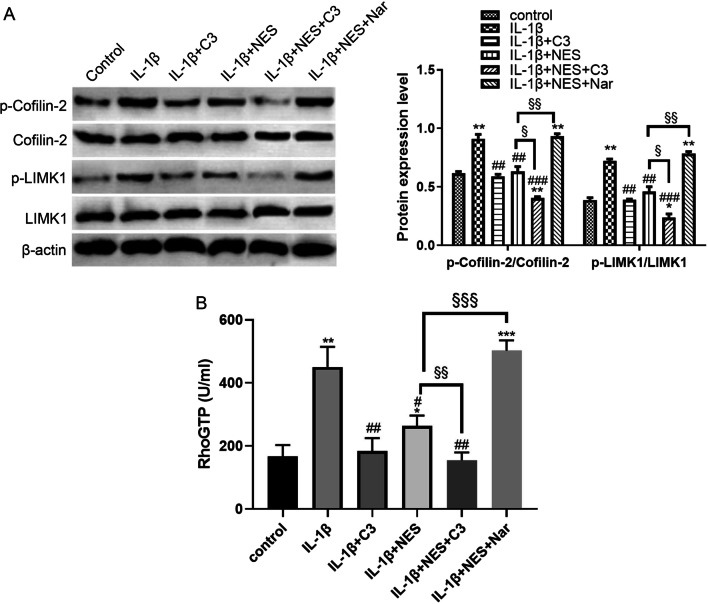
RhoA/ROCK activator weakened the inhibitory effect of nesfatin-1 in RhoA/ROCK pathway in OA chondrocytes. **A** The expression of RhoA/ROCK downstream proteins (Cofilin-2, p-Cofilin-2, LIMK1 and p-LIMK1) in OA chondrocytes in different groups determined by western blot and quantified by ImageJ. **B** The activity of RhoGTPase in OA chondrocytes in different groups determined by RhoGTP ELISA kit. Data are expressed as means ± SD of 3 independent experiments. **p < 0.01 compared with control group; ^#^*p* < 0.05, ^##^*p* < 0.01, compared with IL-1β group; ^§§^*p* < 0.01, ^§§§^*p* < 0.001 compared with IL-1β + NES group

In order to confirm the specific role of the RhoA/ROCK pathway in conjunction with nesfatin-1, we analyzed and compared cytoskeleton integrity and downstream target proteins after administration of RhoA/ROCK activator (Narciclasine, Nar, 50 nM) or inhibitor (C3 exoenzyme, C3, 300 nM) with or without NES. Firstly, western blot assay was done to investigate the expression of RhoA/ROCK downstream proteins in different treatments (Fig. 6A). Compared with OA chondrocytes, C3 and NES treatment lowered phosphorylation levels of Cofilin-2 and LIMK1 in OA chondrocytes, and C3 strengthened the effect of NES on RhoA/ROCK signal activation, whereas treatment with Nar presented the opposite outcome, in that Nar weakened the effect of NES. These results collectively demonstrate that the RhoA/ROCK pathway participates in regulating the course of OA and is inhibited by nesfatin-1. Furthermore, a similar trend was observed in RhoGTP levels of the different groups (Fig. 6B). These results corroborate the role that NES plays in OA chondrocytes via RhoA/ROCK signaling.

**Fig. 7:**
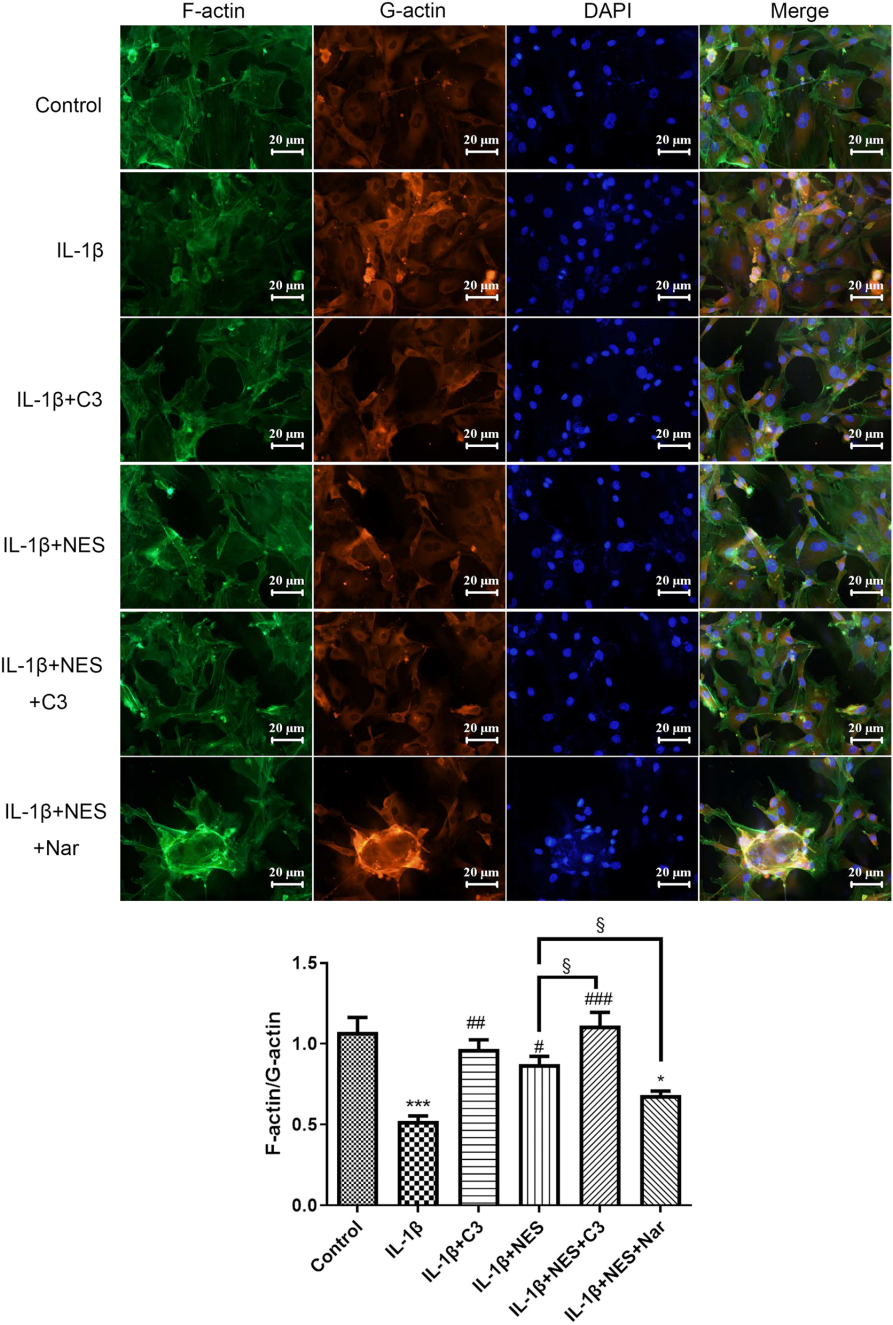
Pre-treatment with nesfatin-1 improved the cytoskeleton integrity by blocking of RhoA/ROCK pathway by immunofluorescence staining of F-actin and G-actin in OA chondrocytes. The quantifications of mean fluorescence intensity were analyzed by ImageJ. Data are expressed as means ± SD of 3 independent experiments. *p < 0.05, ***p < 0.001 compared with control group; ^#^*p* < 0.05, ^##^*p* < 0.01, ^###^*p* < 0.001 compared with IL-1β group; ^§^*p* < 0.05 compared with IL-1β + NES group

A remarkably lower F-actin/G-actin proportion was revealed in OA chondrocytes (IL-1β group) as shown in Fig. 7, compared to the control group, indicating cytoskeleton destruction in the OA group. When pre-incubated with NES (IL-1β + NES group), C3 (IL-1β + C3 group) or NES + C3 (IL-1β + NES + C3 group), the ratios were significantly elevated compared to IL-1β group, validating that NES and C3 inhibited the RhoA/ROCK pathway to improve cytoskeleton integrity, and C3 was able to intensify the effect of NES. On the contrary, RhoA activator Nar (IL-1β + NES + Nar group) weakened the effect of NES (IL-1β + NES group) on cytoskeleton integrity. The rescue experiment unquestionably revealed the effect of RhoA/ROCK on chondrocyte cytoskeleton integrity.

**Fig. 8:**
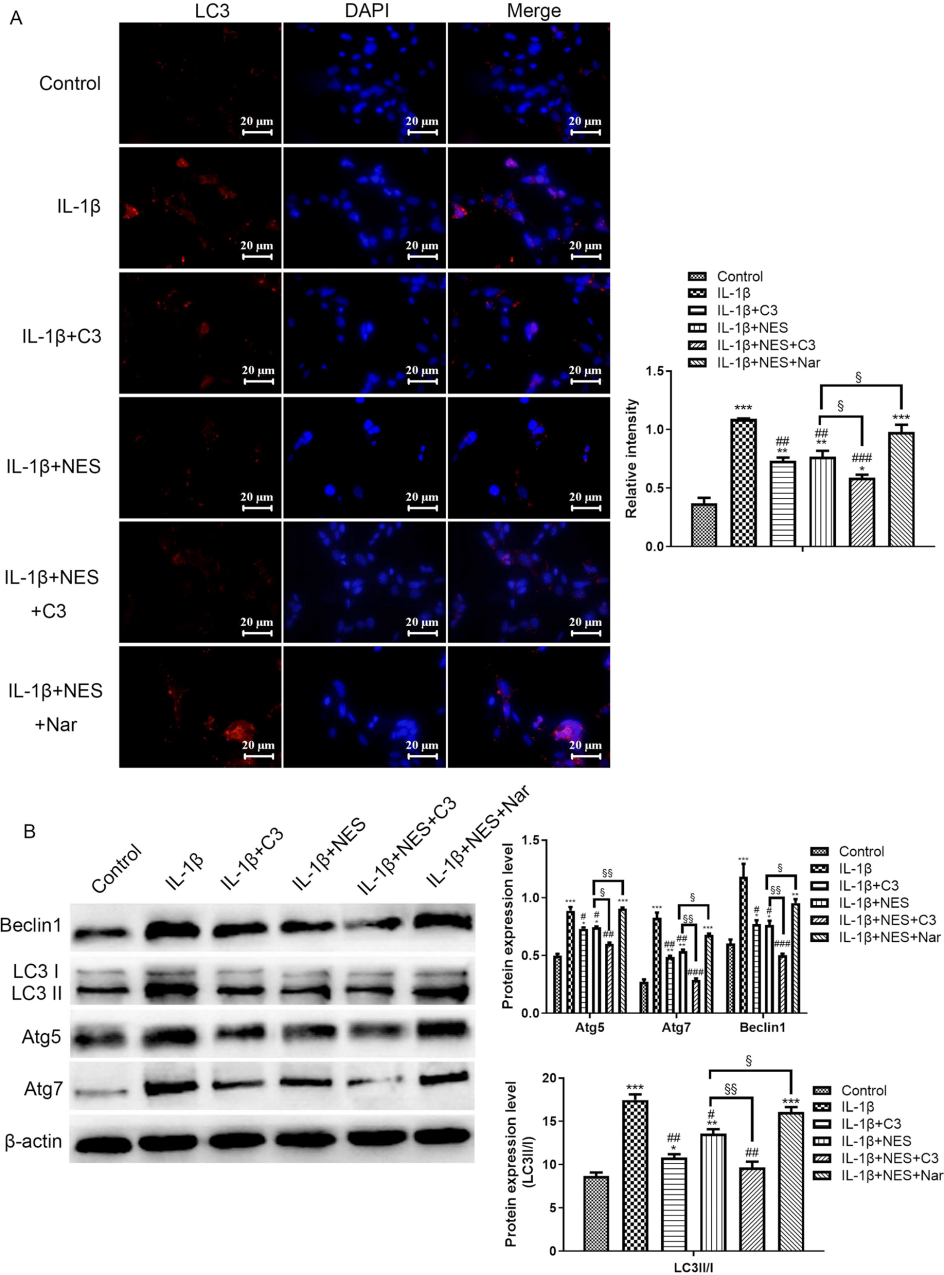
Pre-treatment with nesfatin-1 suppressed the excessive autophagy level by blocking of RhoA/ROCK pathway in OA chondrocytes. **A** Immunofluorescence staining of LC3 and the quantification of mean fluorescence intensity was analyzed by ImageJ. **B** The expression levels of autophagy correlated proteins including Atg5, Atg7, Beclin1 and LC3 by western blot and analyzed by ImageJ. Data are expressed as means ± SD of 3 independent experiments. *p < 0.05, **p < 0.01, ***p < 0.001 compared with control group; ^#^*p* < 0.05, ^##^*p* < 0.01, ^###^*p* < 0.001 compared with IL-1β group; ^§^*p* < 0.05, ^§§^*p* < 0.01 compared with IL-1β + NES group

To further elucidate the role that the RhoA/ROCK pathway played in the excessive autophagy observed in OA chondrocytes at the 24 h timepoint, immunofluorescence staining on LC3, as shown in Fig. 8A, revealed that chondrocytes treated with IL-1β demonstrated excessive LC3 compared with normal chondrocytes (control). C3 and NES pre-treatment significantly reduced the autophagy levels in OA chondrocytes. C3 strengthened the inhibitory effect of NES on autophagy, while Nar displayed the opposite effect, proving that RhoA/ROCK played a regulatory role in mediating the extent of autophagy at the 24 h timepoint. Similarly, C3 and NES significantly decreased the levels of other autophagy correlated proteins, specifically Atg5, Atg7, Beclin1 (Fig. 8B). C3 and NES also lowered the LC3II/I ratio in OA chondrocytes, suggesting that the RhoA/ROCK pathway participated in regulating autophagy. From these results, we can confirm that there is a relationship among cytoskeleton remodeling, RhoA/ROCK pathway and autophagy, concurrently affecting the course of OA.

### Nesfatin-1 significantly precluded OA exacerbation in vivo via inhibiting the activation of RhoA/ROCK pathway

**Fig. 9:**
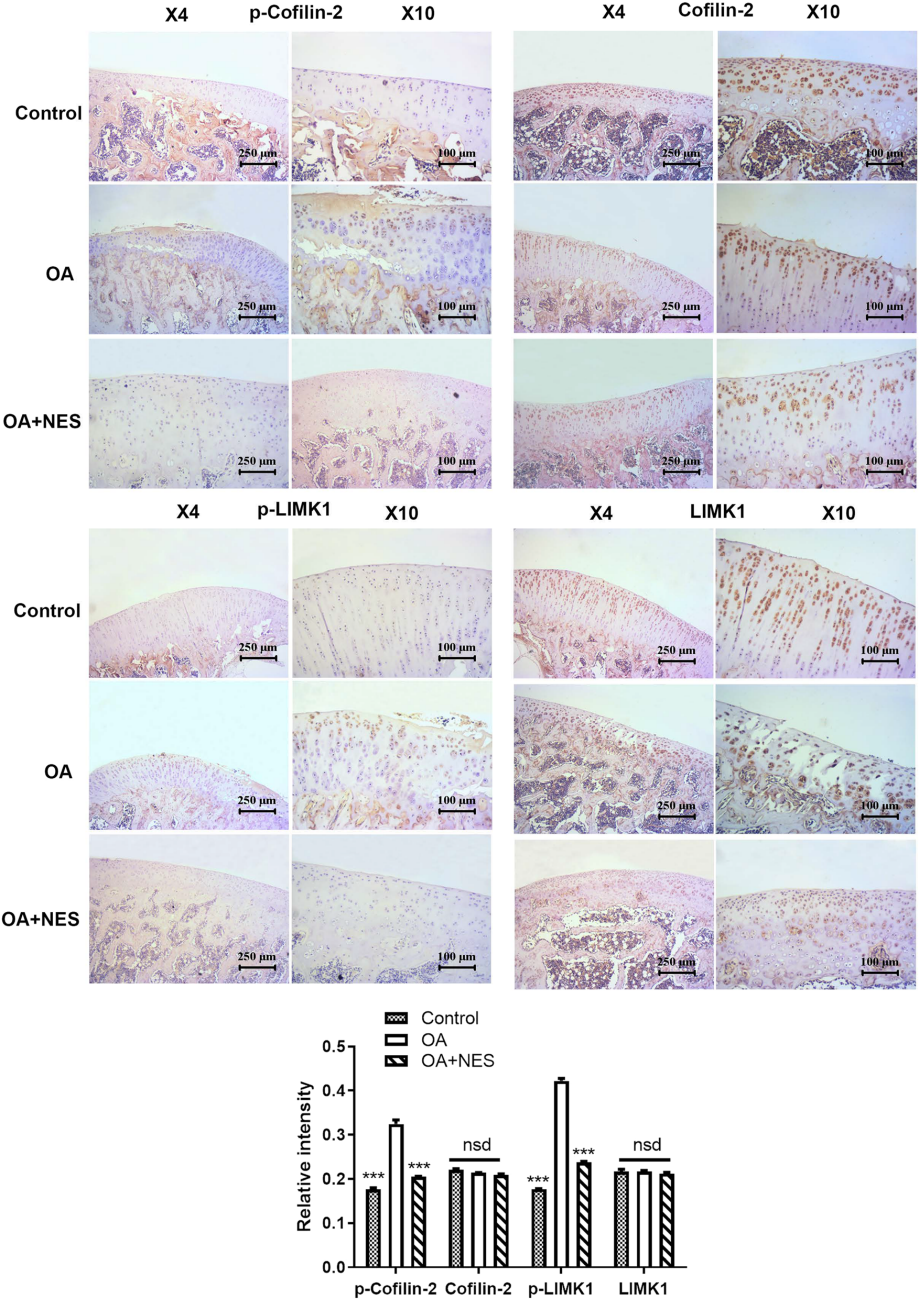
Nesfatin-1 significantly precluded OA exacerbation in vivo via inhibiting the activation of RhoA/ROCK pathway. Immunohistochemical staining of the downstream proteins of RhoA/ROCK pathway including Cofilin-2, p-Cofilin-2, LIMK1 and p-LIMK1 in different groups. ***p < 0.001 compared with OA group; nsd means no significant difference

Immunohistochemical staining of downstream proteins of the RhoA/ROCK pathway, including Cofilin-2, phosphorylated Cofilin-2, LIMK1 and phosphorylated LIMK1, was conducted on in vivo samples, as shown in Fig. 9. Consistent with the results presented in OA chondrocytes, the cartilaginous tissues of OA rats treated with nesfatin-1 showed lower expression of phosphorylated Cofilin-2 and phosphorylated LIMK1 when compared with OA rat tissues without any treatment, indicating that nesfatin-1 also inhibited the RhoA/ROCK pathway in vivo, and thus ameliorated osteoarthritis.

## Discussion

Osteoarthritis has become one of the most common chronic disabilities causing diseases with a rising incidence mostly affecting older populations [25, 26]. Current researches have been able to unfold the potential that the adipokine nesfatin-1 holds in remodeling cytoskeleton and preventing cell apoptosis, as well as its anti-inflammatory effects [27–29]. Moreover, nesfatin-1 has been reported to be a positive regulatory factor in precluding the course of OA. Our previous study detected the presence of nesfatin-1 in joint tissue, serum and chondrocytes of OA patients. In this study, we used IL-1β to induce OA [30] and found that nesfatin-1 significantly optimized cytoskeleton integrity that has been destroyed by IL-1β, proving that nesfatin-1 is capable of protecting chondrocytes heading for OA [20]. However, the specific molecular mechanism of nesfatin-1 in protecting chondrocytes from OA remains largely unknown. Previous studies demonstrated that the RhoA/ROCK pathway is regulated by nesfatin-1 in ovarian epithelial carcinoma [31], which gave us grounds to hypothesize that nesfatin-1 mediates the course of OA via the RhoA/ROCK pathway.

Abnormal autophagy has been reported in OA patients, and there is a pathological correlation between autophagy levels and OA [7, 32]. This study first examined the expression of autophagy-related proteins, including Beclin1 and p62. Results demonstrated that after 24 h of IL-1β induction, Beclin1 presented the highest expression quantities, and p62 presented the lowest expression in OA chondrocytes (Fig. 1), suggesting that excessive autophagy was observed in OA chondrocytes at 24 h, which could lead to irreversible damage and apoptosis. Subsequently, cytoskeleton destruction was demonstrated with immunofluorescence which showed the ratio of F-actin to G-actin in OA chondrocytes. After pre-treatment with 10 ng/ml of nesfatin-1 at 10 min, 20 min and 30 min, the cytoskeleton of OA chondrocytes significantly and successively improved. As can be seen at 30 min of nesfatin-1 pre-incubation, cytoskeleton integrity of OA chondrocytes was impressively restored to a state very similar to that of normal chondrocytes (Fig. 2). Based on mRNA, protein and immunofluorescence levels, it was observed that pre-treatment with nesfatin-1 significantly reduced autophagy at 24 h after IL-1β induction in chondrocytes (Figs. 3, 4). RhoA/ROCK signaling has been reported to be closely associated with cytoskeleton remodeling [33, 34]. Western blot and RhoGTP ELISA assay were conducted to investigate the effect of nesfatin-1 on RhoA/ROCK pathway. Our results revealed that pre-treatment with nesfatin-1 inhibited the RhoA/ROCK pathway in OA chondrocytes and thereby improved the course of OA (Fig. 5).

The exact mechanism as to which nesfatin-1 acts on the RhoA/ROCK pathway in OA has not yet been studied thoroughly to this day. Our study on the effects of nesfatin-1 aimed to shed light on how it participates in mediating the development of OA through remodeling of the cytoskeleton via the RhoA/ROCK pathway. In our study, we used the RhoA activator Narciclasine and RhoA inhibitor C3 exoenzyme to explore the action of nesfatin-1 in the RhoA/ROCK pathway during OA progression. Our results collectively prove the involvement of NES in OA progression, especially through the RhoA/ROCK pathway. All results collected indicate that nesfatin-1 enhanced cytoskeleton integrity (Fig. 7) as well as inhibited excessive autophagy level in OA chondrocytes. Furthermore, OA rats given nesfatin-1 showed down-regulated levels of phosphorylated Cofilin-1 and LIMK1, thus confirming that nesfatin-1 precluded the activation of RhoA/ROCK pathway in vivo (Fig. 9). This effect of NES is similar to that of C3, and we found that we could rescue this effect by adding the RhoA activator Nar.

From a clinical standpoint, it has been made apparent that nesfatin-1 level is significantly higher in OA patients [20] compared with normal tissues, and although previous studies have demonstrated the anti-inflammatory and anti-apoptotic functions of nesfatin-1 in OA chondrocytes [18], the therapeutical prospects of nesfatin-1 require further exploration. Consequently, we report for the first time that nesfatin-1 plays a crucial role in preventing the exacerbation of osteoarthritis by restoring cytoskeleton integrity of chondrocytes and suppressing excessive autophagy level via inhibiting the RhoA/ROCK pathway. Our discovery provides a promising therapeutic strategy for osteoarthritis.

In conclusion, we demonstrated that nesfatin-1 suppressed excessive autophagy and improved chondrocyte cytoskeleton integrity in OA cartilage through our in vivo and in vitro experiments. Nesfatin-1 was found to exert favorable effects through inhibiting the activation of RhoA/ROCK pathway both in vitro and in vivo. These results present a potential therapeutical strategy for OA.

## Data Availability

The data that support the findings of this research are available from the corresponding author upon reasonable request.
